# Perceived stress and mental health in perimenopausal women: a serial mediation study of psychological distress and social support

**DOI:** 10.3389/fpsyt.2026.1730812

**Published:** 2026-05-20

**Authors:** Tingting Ruan, Ying Yuan, Yiwei Li, Lijia Hou, Xuyan Liu, Shuling Ye, Jing Lu, Xue Tang, Yang Liu

**Affiliations:** Department of Gynecology, Deyang People’s Hospital, Deyang, Sichuan, China

**Keywords:** mental health, perceived stress, perceivedsocial support, perimenopause, psychological distress

## Abstract

**Background:**

The perimenopausal phase is associated with a significantly higher prevalence of mental health disorders in women, with stress perception emerging as a pivotal risk factor. However, the psychological and social mechanisms through which stress perception influences women’s mental health during this period remain to be fully elucidated. This study aims to use a stress process model to examine how social support mediates the link between stress perception and psychological symptom severity during perimenopause.

**Methods:**

A cross-sectional survey design was used, and 549 Chinese perimenopausal women were surveyed through face-to-face questionnaires. The survey employed the Chinese Perceived Stress Scale, Kessler Psychological Distress Scale, Perceived Social Support Scale, and Psychological symptom severity (BSRS-5) to evaluate participants’ psychological symptom severity. The researchers used SPSS 26.0 for related analyses, PROCESS macro software for regression analyses, and applied the Bootstrap method to assess mediating effects.

**Results:**

The findings of the study indicate that perceived stress, psychological distress, and psychological symptom severity (BSRS-5) are significantly and positively correlated, and perceived social support is significantly and negatively correlated with these variables (*P* < 0.01). The study reveals that perceived stress significantly increases psychological symptom severity scores(BSRS-5) (effect size=0.493, 59.60%) after adjusting for confounding variables. Additionally, psychological distress and perceived social support independently mediate this relationship (effect sizes=0.204, 24.67% and 0.101, 12.21%, respectively). Additionally, perceived stress indirectly affects psychological symptom severity(BSRS-5) through the chain-mediated mediating pathway of “psychological distress → perceived social support” (effect size = 0.030, percentage = 3.62%).

**Conclusion:**

Stress can directly increase psychological symptom severity in perimenopausal women and indirect effects can be observed through mediating factors such as psychological distress, perceived social support, and the chain-mediated relationship between these two elements. Thus, reducing symptom severity is essential for improving mental health. The study indicates that enhancing the mental health of this group requires a multifaceted approach. This approach should focus on the alleviation of psychological distress and the promotion of social support systems. This will effectively disrupt the cycle of stress and psychological distress.

## 1 Introduction

Perimenopause is a major global public health issue affecting the health of middle-aged women, as evidenced by the prevalence of adverse physical and mental health outcomes experienced by over 80% of this demographic (1), this seriously affects quality of life and mental health (2, 3). By 2030, China will have over 280 million women aged 50 and over, while the global total is expected to reach 1.2 billion (4). The unique stressors faced by this group—including, but not limited to, career development, family responsibilities, changes in self-image, and anxiety about aging—have caused their perceived stress levels to rise significantly. These factors may constitute key risk factors for their mental health (5, 6). The prevailing public health system places a predominant emphasis on the management of physical symptoms in perimenopausal women (7–9), a conspicuous absence in the system is a systematic approach to psychosocial support resources. A substantial body of scientific evidence has conclusively demonstrated significant associations between perimenopausal physical and psychological symptomatology and adverse health consequences, including heightened susceptibility to cardiovascular disease (8), sleep disturbances (10) and cognitive deterioration (11). The significant implications highlight the need to investigate the mechanisms through which perceived stress impacts mental health in this group to create effective interventions.

### 1.1 The relationship between perceived stress and mental health

Mental health refers to an individual’s cognitive, emotional, and social well-being, including abilities like emotional regulation, stress management, and social adaptation (12). Among individuals experiencing perimenopause, mental health issues are of particular concern. During this phase, women experience neuroendocrine disturbances triggered by fluctuating estrogen levels, significantly heightening their sensitivity to stressors (13, 14). Perceived stress, in this context, is defined as a core stressor, reflecting an individual’s subjective assessment that life demands exceed their coping capacity (15). In order to develop a more profound comprehension of the manner in which perceived stress influences mental health, the cognitive appraisal theory of stress offers a theoretical framework for its elucidation (16). This theory posits that stress is not solely triggered by external environmental events, but rather arises from a dynamic interactive process between the individual and their environment. When an individual perceives environmental demands as threatening or challenging while simultaneously assessing their own coping resources as insufficient, perceived stress emerges. This experience of stress triggers a series of emotional, cognitive, and behavioral responses, ultimately impacting physical and mental health outcomes (17). Research indicates that perceived stress significantly adversely affects mental health (18, 19). Accordingly, it is hypothesized that perceived stress can significantly and positively predict the severity of psychological symptoms, as evidenced by both the theoretical framework and the empirical evidence. Despite the substantiation of the relationship between the two by research, the stress source of perimenopausal women through which internal psychological and social path ultimately affects the health outcome warrants further investigation by researchers.

### 1.2 The mediating role of psychological distress

Psychological distress refers to a range of negative emotional reactions experienced under stress, primarily presenting as symptoms of depression, anxiety, and somatization (20, 21). In perimenopausal women, fluctuations in neuroendocrine systems result in diminished stability within their emotional regulation systems, rendering this demographic vulnerable to psychological distress (14). Nolen-Hoeksema’s “response style theory” offers an explanatory framework for understanding how stress causes and maintains psychological distress (22), The theory posits that individuals adopt different coping response styles after experiencing a stressful event, with rumination—defined as a pattern of thinking that passively and repeatedly focuses on the symptoms of one’s distress and their possible causes and consequences—being the central mechanism that leads to and prolongs negative moods such as depression and anxiety. A high degree of rumination in individuals can result in a self-perpetuating cycle of stress, with the onset of stress prompting rumination, which, in turn, exacerbates negative cognition and emotional distress. This, in turn, impairs problem-solving and social functioning, thereby becoming a new source of stress. This dynamic perpetuates psychological distress, leading to its persistence and exacerbation. In accordance with this theoretical framework, empirical research has substantiated the pivotal function of psychological distress in the stress-health nexus (23). Cross-sectional studies indicate that perceived stress indirectly heightens psychological distress by enhancing the tendency to ruminate (24, 25). Research indicates that interventions like positive thinking training can significantly alleviate the adverse impact of stress on psychological distress by decreasing ruminative thinking (26–28). This further supports the inverse mediating role of psychological distress. Drawing from the theoretical framework and empirical evidence, we hypothesize that psychological distress mediates the link between perceived stress and mental health. In other words, perceived stress exacerbates an individual’s poor coping style (e.g., rumination) by stimulating and maintaining their psychological distress levels. This, in turn, leads to a deterioration in psychological symptom severity.

### 1.3 The mediating role of perceived social support

Perceived social support refers to the assistance and support that an individual subjectively perceives from their social networks (29). Cohen and Wills’ “stress buffering model” serves as a fundamental theoretical basis for understanding how social support influences the stress-health relationship. The model highlights that increased social support mitigates the adverse effects of stress, leading to decreased anxiety, depression, and physiological disease risk (30). Previous studies have also confirmed the protective role of perceived social support in mental health (31, 32). In a cross-sectional study of perimenopausal women, perceived stress was significantly and negatively correlated with perceived social support (33); perceived social support also showed a significant negative correlation with depressive and anxiety symptoms (34). Research indicates that perimenopausal women undergoing social support enhancement training exhibit reduced stress-related psychological distress compared to a control group (35). We propose that perceived social support mediates the link between perceived stress and psychological symptom severity, grounded in theoretical and empirical evidence. Perceived stress adversely affects mental health both directly and indirectly by diminishing perceived social support.

### 1.4 The sequential mediation of psychological distress and perceived social support in a chain-like process

The analyses confirmed that psychological distress and perceived social support independently mediate the effects. However, stress’s impact on psychological symptom severity often occurs through chained pathways involving multiple variables. Pearlin et al.’s Stress Process Model (SPM) provides a systematic theoretical framework for understanding these pathways (36). The SPM explains how stress creates chain reactions among social structures, individual resources, and health outcomes. The SPM is divided into three levels: stressors, mediating/moderating resources, and outcomes. The model highlights that resources like social support can mitigate or alter the effects of stress on health. It categorizes stressors as primary initial stressors and secondary stressors (36). Based on the SPM framework, we propose a chain mediation model. Perceived stress is the main initial stressor that first triggers internal psychological distress, forming a secondary stressor. This negative emotional and cognitive state then undermines external social support resources, forming a stressor that ultimately leads to a decline in mental health. Some indirect evidence supports this chain’s plausibility. For example, a longitudinal study indicates that perceived stress predicts a decline in social support levels by increasing psychological distress (37). Another study revealed that, among individuals with more severe depressive symptoms, perceived stress significantly strengthens the negative predictive effect on social support (38). This study hypothesizes that perceived stress will have an indirect negative effect on mental health through the chain-mediated pathway of “psychological distress → perceived social support” within the SPM framework. Although this chain pathway is theoretically valid, further research is needed to directly target the perimenopausal population.

### 1.5 Current research

Previous research has explored the connections among perceived stress, psychological distress, perceived social support, and psychological symptom severity, focusing solely on individual mediation pathways between these factors. These studies have failed to test the chain mediation pathway of “psychological distress–perceived social support” within an integrated theoretical framework. This makes it difficult to fully reveal the underlying processes through which perceived stress affects psychological symptom severity. Furthermore, specialized research on women during the transitional period of perimenopause is scarce, and this group’s unique physiological, psychological, and social challenges have not received sufficient attention. Therefore, this study uses the SPM as its overarching theoretical framework and aims to construct a chained mediation model to validate the underlying process through which perceived stress influences the mental health of perimenopausal women. This research aims to inform the development of intervention measures to more effectively maintain and promote the psychological symptom severity of women during perimenopause. We hypothesized that:

H1: Perceived stress significantly and positively predicts the severity of psychological symptoms.

H2: Psychological distress acts as a mediator between perceived stress and psychological symptom severity.

H3: Perceived social support acts as a mediator between perceived stress and psychological symptom severity.

H4: Perceived stress indirectly affects psychological symptom severity through the chain-mediated pathway of “psychological distress → perceived social support”.

## 2 Materials and methods

### 2.1 Study design, procedure, and participants

This cross-sectional study was conducted in Sichuan, China. Data collection commenced on 10 February 2025 and concluded on 2 September 2025.

#### 2.1.1 Sampling frame and recruitment strategy

This study used a convenience sampling method. The sampling frame was the gynecology and perimenopause specialty clinics of four tertiary hospitals in Chengdu and Deyang, Sichuan Province. The recruitment process is as follows:(1) Identification: Trained research personnel contact potential participants in the outpatient waiting area; (2) Preliminary screening: A brief oral screening is performed to confirm preliminary eligibility based on two key criteria: age (40–60 years old) and self-reported menstrual changes indicating perimenopause; (3) Detailed explanation and informed consent: Eligible women are then invited to a private area where the research personnel provide a detailed explanation of the study and obtain their written informed consent. All study participants should follow the following inclusion and exclusion criteria, with the inclusion criteria as follows: (1) Age 40–60 years; (2) Meets basic criteria for perimenopause diagnosis: menstrual cycle changes (lengthening or shortening) >7 days, or cessation of menstruation for <12 months; (3) Informed consent obtained, with ability to cooperate well in questionnaire completion. Exclusion Criteria: (1) Communication barriers due to cognitive impairment, psychiatric disorders, or hearing/vision abnormalities; (2) Severe physical illnesses such as malignant tumors; (3) Non-natural menopause (e.g., surgical, chemoradiation-induced); (4) Major stressful events within the past 6 months (e.g., bereavement, divorce, significant financial loss). This study employed an online questionnaire (https://www.wjx.cn/wjx/design/previewmobile.aspx?activity=316382414&s=1). Data were collected using an online questionnaire platform administered in a face-to-face setting. Prior to beginning, researchers explained the method and precautions for filling out the questionnaire. Participants, having comprehended the research objectives, signed informed consent forms and completed the questionnaire anonymously. To ensure inclusivity, participants who were illiterate or had difficulty reading were not excluded. For these participants, trained interviewers administered the questionnaire verbally in a standardized, face-to-face interview format. The interviewers read each question and the corresponding response options exactly as worded, without interpretation or elaboration. To minimize interviewer bias and preserve data fidelity, the interviewers recorded the participants’ chosen responses verbatim on the questionnaire form. This procedure ensured that all data, whether self-reported or interviewer-assisted, were collected with maximum consistencyAll researchers received standardized training. Questionnaires were completed and collected on-site. Before submitting them, interviewers checked each questionnaire immediately for omissions or obvious logical errors. Any issues were resolved on the spot. Each questionnaire took 10–15 minutes to complete. Based on preliminary pre-surveys, questionnaires that were completed in under 300 seconds or that had the same answer selected for every question were deemed invalid.

#### 2.1.2 Sample size and power analysis

To determine the minimum sample size required to test the chained mediation model proposed in this study, we conducted a power analysis using Monte Carlo simulations with the online tool (https://schoemanna.shinyapps.io/mc_power_med/). This method is applicable to complex models involving multiple indirect effects, for which traditional power formulas are not applicable. The simulation parameters were obtained from a pre-survey of 50 peri-menopausal women and the expected path coefficients are as follows: perceived stress to psychological distress (a_1_) = 0.649; psychological distress to perceived social support (d) = -0.353; perceived stress to perceived social support (a_2_) = -0.581; psychological distress to psychological symptom severity (b_1_) = 0.588; perceived social support to psychological health symptoms (b_2_) = -0.425. Given a significance level (α) of 0.05 and a target statistical power (1 - β) greater than 0.90, the simulation results (after 5, 000 iterations of resampling) indicate that a sample size of N = 362 is sufficient to detect the total indirect effect in the proposed model.Considering the possibility of invalid questionnaires and sample loss during data collection, we increased the target sample size by 20%, resulting in the planned sample size of N = 434. We initially approached 644 women, of whom 40 did not meet the inclusion criteria, 31 were excluded due to short response times (<300 seconds), and 24 were excluded for providing identical answers to all items. Consequently, the final sample size for analysis was N = 549, exceeding the minimum required sample size and ensuring adequate statistical power.

#### 2.1.3 Ethical approval

This study was approved by the Ethics Committee of the People’s Hospital of Deyang City, China (approval number: 202404125K01). All participants provided written informed consent.

### 2.2 Measures

#### 2.2.1 Demographic information questionnaire

The instrument was designed by researchers to collect participants’ demographic characteristics and potentially relevant covariate information. The content includes: (1) Age; (2) Residence; (3) Education; (4) Marital status; (5) Children count; (6) Grandchild caregiving; (7) Chronic disease; (8) Menopausal status; (9) Occupation; (10) Monthly income per capita; (11) Exercise frequency; (12) Social activity participation; (13) Family attitudes towards social interactions.

#### 2.2.2 Chinese perceived stress scale, CPSS

The Chinese version of the 14-item Perceived Stress Scale, originally developed by Cohen et al. (15), and translated by Yang et al. (39), was utilized to evaluate individuals’ perceived stress levels related to life events in the past month. The scale under consideration comprises two dimensions: a sense of helplessness, which is measured using six items, and a feeling of tension, which is measured using four items. The scale employs a 5-point Likert format, with values from 0 (‘Never’) to 4 (‘Always’). Notably, four of the scale’s items are reverse-scored. The total score ranges from 0 to 70 points and is categorized into four levels: 14–28 points indicate low perceived stress, 29–42 points represent moderate perceived stress, 43–56 points signify high perceived stress, and 57–70 points denote very high perceived stress. Conversely, higher scores on the scale are indicative of elevated stress levels. The scale’s Chinese version has shown sufficient reliability and validity within Chinese populations. The scale’s Cronbach’s α coefficient was found to be 0.972 in this study.

#### 2.2.3 Kessler psychological distress scale, K10

The scale was initially developed by Kessler (40), and subsequently adapted by Chinese researcher Zhou Chengchao (41), for use in assessing the extent of non-specific symptoms, including anxiety and depression, in individuals. The scale comprises 10 items, each rated on a 5-point Likert scale from 1 (none) to 5 (all the time).

The scale’s total score ranges from 10 to 50, categorized into four levels: 10–15 for almost no distress, 16–21 for mild distress, 22–29 for moderate distress, and 30–50 for severe distress. A higher score indicates a greater level of psychological distress. The scale’s Cronbach’s alpha coefficient was found to be 0.969 in this study.

#### 2.2.4 Perceived social support scale, PSSS

Adapted from Zimet et al. (42), revised and adapted for Chinese by Qianjin Jiang (43). The scale under consideration comprises 12 items, which are designed to assess three distinct dimensions: family support, friend support, and support from other significant others. A 7-point Likert scale was utilized, with options from 1 (‘Strongly Disagree’) to 7 (‘Strongly Agree’). The total score ranges from 12 to 84 points and is categorized into four levels: 12–36 points indicate low social support, 37–60 points represent moderate social support, and 61–84 points denote high social support. Conversely, higher scores on the scale indicate a greater perceived level of social support. The Chinese version of the scale has demonstrated adequate reliability and validity. The scale demonstrated a Cronbach’s α coefficient of 0.986 in this study.

#### 2.2.5 Brief symptom rating scale, BSRS-5

The Brief Symptom Rating Scale-5 (BSRS-5) was developed as a concise screening tool for psychological symptom severity, it serves as the primary outcome measure in this study, operationalizing the construct of psychological symptom severity (44). The scale consists of five highly correlated items chosen from the BSRS-50. Furthermore, the scale includes an extra question. Specifically, the BSRS-5 assesses the following symptoms: difficulty sleeping, feelings of annoyance or anger, depressed mood, feelings of inferiority toward others, and suicidal thoughts. The total score is adjusted to a 0–24 scale using a 5-point Likert scale, where 0 represents ‘not at all’ and 4 represents ‘very much’. Scores on this scale range from 0 to 24, with higher scores indicating greater severity of psychological symptoms. For clinical reference, a total score below 3 is considered indicative of no significant psychological distress, scores between 3 and 6 suggest mild to moderate distress, and scores above 6 suggest a potential need for professional mental health evaluation. This scale has been extensively utilized in population studies due to its brevity and good psychometric properties. In this study, the BSRS-5 demonstrated a Cronbach’s alpha coefficient of 0.89.

### 2.3 Statistical analysis

The data analysis was conducted using SPSS 26.0 software, equipped with its PROCESS plugin, version 4.1. Initially, all collected data underwent a thorough cleansing and structuring process. A series of normality tests were conducted on the quantitative data. Data with a normal distribution were represented as mean ± standard deviation. For the purpose of making comparisons between two groups, an independent samples t-test was employed. A one-way analysis of variance (ANOVA) was employed for comparisons involving three or more groups. For data not following a normal distribution, values were represented as M (P25, P75). The Mann–Whitney U test was used for two-group comparisons, and the Kruskal–Wallis H test was applied for comparisons involving three or more groups. Statistical tests were conducted using a two-tailed approach with a significance threshold of α=0.05. The internal consistency reliability of each scale was assessed using Cronbach’s α coefficient, with values above 0.70 signifying sufficient reliability. To analyze the pairwise relationships among perceived stress, psychological distress, perceived social support, and mental health, Pearson correlation was used for normally distributed data, while Spearman rank correlation was applied for skewed data. The correlation coefficient, denoted by r, was employed to evaluate the magnitude and orientation of associations between variables. To assess potential common method bias (CMB), a single-factor confirmatory factor analysis (CFA) model was used, loading all observed variables onto one latent variable to evaluate model fit. A single-factor model with adequate fit suggests the presence of significant CMB, while a poor fit indicates negligible CMB. Before conducting regression analysis, we assessed multicollinearity among the predictor variables (perceived stress, psychological distress, perceived social support) and covariates by examining the variance inflation factor (VIF). Typically, a VIF value exceeding 10 (or a tolerance below 0.10) indicates severe multicollinearity, which may undermine the stability of regression estimates. The chain mediation analysis utilized Hayes’ PROCESS 4.1 macro (Model 6). A bias-corrected nonparametric percentile bootstrap approach, utilizing 5, 000 resamples, was used to determine 95% confidence intervals.

## 3 Results

### 3.1 Assessment of common method bias

A single-factor confirmatory factor analysis (CFA) was employed to examine common method bias. The results showed that the single-factor model’s fit indices were below conventional standards, with CFI = 0.573, TLI = 0.551, and RMSEA = 0.188, whereas typical requirements are CFI and TLI ≥ 0.90 and RMSEA ≤ 0.08. Therefore, the poor fit of the single-factor model indicates no severe common method bias in the data. The study’s data quality is satisfactory, with negligible common method bias, ensuring no significant impact on subsequent empirical analyses.

### 3.2 Univariate analysis of mental health in perimenopausal women

This study included 549 participants, with the majority aged between 51 and <56 years (28.4%, n=156) or 46 and <51 years (27.9%, n=153). Most resided in urban areas (56.8%, n=312), were married with a partner (89.4%, n=491), with spouses in good health (81.4%, n=447) and one child (72.0%, n=396). A predominant proportion of the sample reported completing no more than secondary education(81.2%, n=446), reported good physical health (78%, n=428), were employed primarily (31.3%, n=172), and had a household monthly income below 4000 yuan (44.8%, n=246). (See **Table 1** for details).

**Table 1:** Univariate analysis of brief symptom rating scores across different characteristics of the study participants (N = 549).

Characteristic	Group	N (%)	BSRS-5 M (P25, P75)	*Z*/*H*	*P*
Age(years)	40-<46	150 (27.3)	3.00 (1.00, 7.00)	0.358	0.949
	46-<51	153 (27.9)	3.00 (1.00, 7.00)		
	51-<56	156 (28.4)	3.00 (1.00, 6.75)		
	56-60	90 (16.4)	3.00 (1.00, 6.25)		
Place of residence	Rural	237 (43.2)	3.00 (1.00, 7.00)	-1.132	0.258
	Urban	312 (56.8)	3.00(1.00, 6.00)		
Education level	High school or below	446 (81.2)	3.00 (1.00, 7.00)	9.391	0.009^**^
	Associate degree	37 (6.7)	2.00 (1.00, 5.50)		
	Bachelor’s degree or higher	66 (12.0)	2.00 (0.00, 4.25)		
Marital status	Married/Partnered	491 (89.4)	3.00 (1.00, 6.00)	-2.817	0.005^**^
	Unmarried/Single	58 (10.6)	5.00 (1.8, 14.00)		
Number of children	0	10 (1.8)	2.50 (0.8, 9.50)	3.392	0.335
	1	396 (72.1)	3.00 (1.00, 6.00)		
	2	136 (24.8)	3.00 (1.00, 7.00)		
	3	7 (1.3)	1.00 (0.00, 3.00)		
Grandchild caregiving	yes	114 (20.8)	4.00 (1.00, 10.50)	2.537	0.281
	no	182 (33.2)	3.00 (1.00, 6.00)		
	Not applicable	253 (46.1)	3.00 (1.00, 7.00)		
Chronic Disease Status	With chronic disease	121 (22.0)	5.00 (2.00, 12.00)	-4.02	0.000^***^
	Without chronic disease	428 (78.0)	3.00 (1.00, 6.00)		
Menopausal status	Early menopausal transition	268 (48.8)	3.00 (1.00, 6.00)	13.221	0.001^**^
	Late menopausal transition	80 (14.6)	5.00 (2.00, 12.75)		
	Early postmenopause	201 (36.6)	3.00 (1.00, 6.00)		
Occupation	Manual laborer	172 (31.3)	3.50 (1.00, 7.00)	9.562	0.215
	On-site employee (non-manual)	99 (18.0)	2.00 (1.00, 5.00)		
	Self-employed/Freelancer	78 (14.2)	4.50 (2.00, 8.00)		
	Professional/Technical worker	37 (6.7)	3.00 (0.00, 6.00)		
	Unemployed/Seeking work	45 (8.2)	3.00 (1.00, 8.00)		
	Full-time homemaker Retiree	65 (11.8)	3.00 (1.00, 11.00)		
	Retired personnel	50 (9.1)	2.00 (1.00, 5.00)		
	Other	3 (0.5)	5.00 (2.00, 10.00)		
Per Capita Monthly Income (¥)	<4000	246 (44.8)	4.00 (2.00, 8.00)	24.148	0.000^***^
	4001-6000	161 (29.3)	3.00 (1.00, 7.00)		
	6001-8000	86 (15.7)	2.00 (1.00, 5.00)		
	8001-10000	40 (7.3)	2.00 (1.00, 5.00)		
	>10000	16 (2.9)	1.50 (0.00, 3.00)		
Exercise frequency	Never/Rarely	198 (36.1)	4.00 (2.00, 12.00)	39.072	0.000^***^
	1–2 times/week	111 (20.2)	3.00 (1.00, 8.00)		
	3–5 times/week	78 (14.2)	2.00 (1.00, 5.00)		
	Almost daily	162 (29.5)	2.00 (1.00, 4.00)		
Participation in social activities	Almost daily	72 (13.1)	2.00 (1.00, 5.75)	10.874	0.012^*^
	≥1 time/week	134 (24.4)	3.00 (1.00, 7.00)		
	1–3 times/month	199 (36.2)	2.00 (1.00, 5.00)		
	Never	144 (26.2)	4.00 (1.00, 12.75)		
Family attitudes toward social interactions**^#^**	Fully supportive	361 (65.8)	2.00 (1.00, 5.00)	35.736	0.000^***^
	Partially supportive	85 (15.5)	3.00 (1.00, 9.00)		
	Neutral	95 (17.3)	6.00 (3.00, 13.00)		
	Opposed	8 (1.5)	12.50 (6.50, 19.25)		

* *P* < 0.05, ^**^P < 0.01, ^***^P < 0.001.

The Z statistic was derived from the Mann-Whitney U test for comparisons between two groups; the H statistic was derived from the Kruskal-Wallis test for comparisons across three or more groups.

BSRS-5, Brief Symptom Rating Scale-5.

**^#^**The “Opposed” group in “Family attitudes toward social interactions” had a very small sample size (n=8, 1.5%). Results for this category should be interpreted with caution due to limited statistical power.

### 3.3 Descriptive statistics and correlations of key variables in perimenopausal women

**Table 2** presents the results of Spearman’s rank correlation analysis conducted on the study variables. The study found a significant positive correlation between perceived stress and both psychological symptom severity (BSRS-5 score) (r = 0.755, p < 0.01) and psychological distress (r = 0.729, p < 0.01). Additionally, a negative correlation was observed between perceived stress and perceived social support (r = -0.732, p < 0.01). The study identified a significant negative correlation between psychological symptom severity (BSRS-5 score) and perceived social support (r = -0.659, p < 0.01). A negative correlation was found between psychological distress and perceived social support (r = -0.650, p < 0.01).

**Table 2:** Correlations among perceived stress, brief symptom rating, psychological distress and perceived socialsupport (N = 549).

Variables	Perceived stress	BSRS-5	Psychological distress	Perceived social support
Perceived stress	1			
BSRS-5	0.755**	1		
Psychological distress	0.729**	0.732**	1	
Perceived social Support	-0.732**	-0.659**	-0.650**	1

** *P* < 0.01.

BSRS-5, Brief Symptom Rating Scale-5.

### 3.4 The mediating influence of perceived social support on the link between psychological distress and perceived stress in perimenopausal women

#### 3.4.1 Regression analysis of the interplay between perceived stress, psychological distress, social support, and BSRS-5

A sequential mediation analysis was conducted using the Bootstrap method, with 95% confidence intervals estimated through 5, 000 resampling iterations. The model featured perceived stress as the independent variable, psychological symptom severity (BSRS-5 score) as the dependent variable, and psychological distress and perceived social support as sequential mediating variables. To control for potential confounders, covariates significantly associated with the outcome variable (p < 0.05) in univariate analysis (**Table 1**) were included. These covariates comprised: educational attainment, marital status, chronic disease status, menopausal status, per capita monthly income, exercise frequency, social activity participation, and family attitudes toward social interaction. The findings of the study demonstrated that perceived stress exhibited a significant and positive association with psychological distress (β = 0.739, P < 0.01) and psychological symptom severity (β = 0.493, P < 0.001).In contrast, perceived stress exhibited a substantial and adverse correlation with perceived social support (β = -0.586, P < 0.001). Psychological distress significantly negatively predicted perceived social support (β = -0.238, P < 0.001) and significantly positively predicted mental health (β = 0.276, P < 0.01).The findings indicated that perceived social support exhibited a substantial negative correlation with psychological distress (β = -0.171, P < 0.001). The results of multicollinearity diagnostics confirm that the variance inflation factor (VIF) values for all predictor variables in the regression model are well below the 10 threshold (ranging from 2.057 to 3.303), indicating that multicollinearity has not compromised the stability of parameter estimates. (See **Table 3**).

**Table 3:** Multilevel regression analysis for serial mediation model.

Variables	Psychological distress		Perceived social Support		BSRS-5 (Symptom severity)	
	β	t	β	t	β	t
Education level	-0.118	-2.198*	-0.004	-0.09	-0.019	-0.472
Marital status	-0.1	-0.983	-0.1	-1.316	0.091	1.217
Chronic Disease Status	0	-0.009	-0.016	-0.277	0.001	0.148
Menopausal status	-0.024	-0.659	-0.019	-0.697	0.003	0.113
Per Capita Monthly Income	0.062	1.829	0.131	5.207***	0.035	1.378
Exercise frequency	0.016	0.575	-0.069	-3.4148*	-0.022	-1.126
Participation in social activities	0.083	2.343*	-0.007	-0.268	0.049	1.869
Family attitudes toward social interactions	-0.134	-2.956**	-0.167	-4.903***	-0.061	-1.786
Perceived stress	0.739	21.072**	-0.586	-16.574***	0.493	11.523***
Psychological distress	-	-	-0.238	-0.7397***	0.276	8.3038**
Perceived social support	-	-	-	-	-0.171	-4.039***
F	59.346**		139.121**		131.834**	
R²	0.497		0.721		0.73	

BSRS-5, Brief Symptom Rating Scale-5. β = Standardized regression coefficient.

*P < 0.05, **P < 0.01, ***P < 0.001.

#### 3.4.2 Examination of psychological distress and perceived social support as mediators in the relationship between perceived stress and BSRS-5

**Figure 1:**
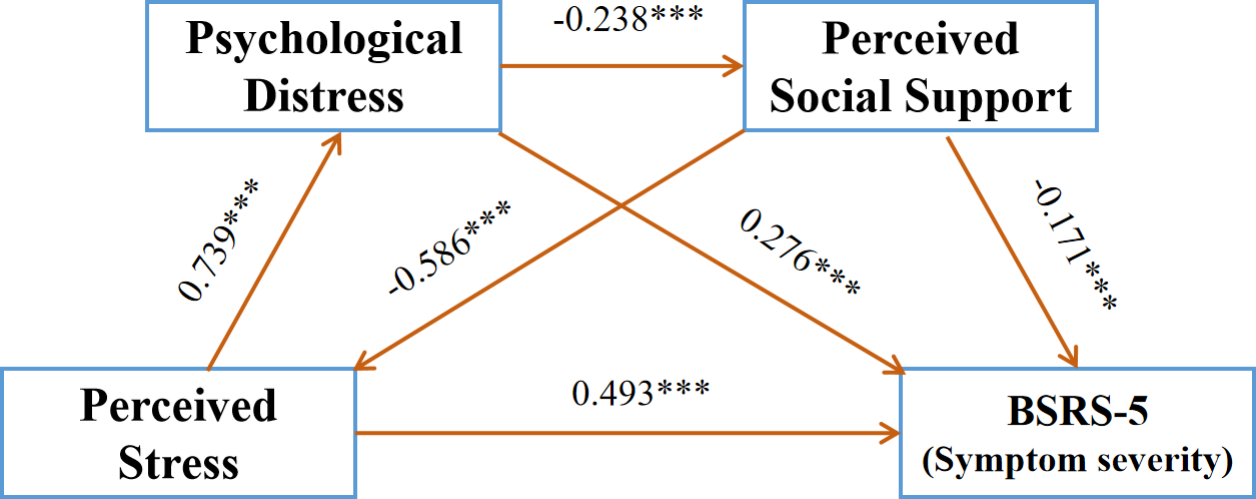
The multiple mediation model (N = 549). ***P < 0.01.

This study developed a chained mediation model to investigate how perceived stress affects mental health, using psychological distress and perceived social support as mediators. The model was assessed through the utilization of Process 4.1 macro Model 6. The findings of the study demonstrated that the confidence intervals for all paths in the model did not include zero, thereby substantiating substantial effects across all paths. The direct impact of perceived stress on psychological symptom severity (BSRS-5 score) was 0.493, representing 59.6% of the overall effect. The study found that perceived distress and social support partially mediated the link between perceived stress and psychological symptom severity, with a total mediation effect of 0.334, accounting for 40.4% of the overall effect. The mediation process can be initiated through three distinct pathways: the first path (effect size = 0.204, representing 24.6% of the total effect), the second path (effect size = 0.101, representing 12.1% of the total effect), and the third path (effect size = 0.03, representing 3.6% of the total effect) were analyzed. **Table 4** details the comprehensive data set, and **Figure 1** illustrates the mediation model.

**Table 4:** Summary of mediation effects on psychological symptom severity (BSRS-5 score).

Path	β	SE	95% CI		Proportion of effect mediated (%)
			UL	LL	
Direct Effect	0.493	0.040	0.409	0.577	59.61
Total Indirect Effect	0.334	0.060	0.230	0.466	40.39
Indirect 1	0.204	0.051	0.122	0.321	24.67
Indirect 2	0.101	0.036	0.031	0.174	12.21
Indirect 3	0.030	0.011	0.010	0.053	3.62
Total Effect	0.827	0.028	0.772	0.883	–

CI, confidence interval; LL, lower limit; UL, upper limit.

Indirect 1 = perceived stress → psychological distress → BSRS-5 score;

Indirect 2 = perceived stress → perceived social support → BSRS-5 score;

Indirect 3 = perceived stress → psychological distress → perceived social support → BSRS-5 score.

## 4 Discussion

### 4.1 Key findings and research significance

This study investigates the mediating roles of psychological distress and perceived social support in the relationship between perceived stress and mental health in perimenopausal women. The stress process model suggests that perceived stress continuously depletes psychological resources, leading to the erosion of social resources, thereby impacting mental health (36). The primary hypotheses of this study were confirmed. The findings suggest that stress perception directly influences mental health (H1) and indirectly affects it via the mediation of Psychological Distress (H2), Perceived Social Support (H3), and the sequential mediation of ‘Psychological Distress → Perceived Social Support’ (H4).This finding suggests that the mental health of perimenopausal women is contingent not only on the management of stress, but also on the acknowledgement and mitigation of stress-induced negative emotions and the disruption of their social support system. This also suggests that we can widely implement universal programs such as mindfulness-based stress reduction and relaxation training in communities and workplaces to help women enhance their stress coping abilities at the source and reduce their perception of stress. Simultaneously, healthcare providers should incorporate screening for depression and anxiety symptoms in perimenopausal women into routine physical examinations. For high-risk individuals identified through screening, timely short-term psychological interventions such as cognitive behavioral therapy should be provided to prevent stress from escalating into psychological distress. Finally, family psychological education can be implemented to guide family members in offering more understanding and supportive companionship. Community care workers should proactively assess the social support networks of these women and encourage them to actively seek help through social skills training. This prevents them from becoming socially isolated due to emotional issues, thereby more thoroughly breaking the vicious cycle.

### 4.2 Perceived stress significantly and positively predicts mental health

This study confirms that perceived stress significantly predicts higher psychological symptom severity, validating Hypothesis H1. Individuals with higher stress levels report more severe psychological symptoms, consistent with findings from other existing research (45, 46). This discovery also supports the “Perceived Stress Theory, “which posits that the stressor itself directly triggers physiological and psychological stress responses in individuals (16). For perimenopausal women, this direct pathway may stem from disruption in the functioning of the neuroendocrine system, decreased sleep quality, or changes in health-related behaviors as a direct result of stress (47–49).The robustness of this direct pathway is supported by long-term longitudinal observational studies. For example, a multi-year follow-up study of perimenopausal women by Woods et al. (50) found that higher levels of current perceived stress during the menopausal transition were significantly associated with more severe and persistent depressive mood trajectories. Importantly, the study also noted no significant differences in hormone levels (e.g., estrone, FSH) among women in different depressive trajectory groups. Collectively, these findings suggest that stress’s impact on mental health may represent a more enduring psychosocial pathway independent of simple endocrine fluctuations. This longitudinal evidence reinforces our findings, suggesting that stress’s negative impact on women’s psychological well-being may persist over time. It underscores the need to prioritize stress management as a core target for early intervention during the perimenopausal period. Based on these results it is recommended that a comprehensive intervention strategy is needed for the mental health of perimenopausal women, which not only needs to intervene in stress-induced Psychological distress, but must also include the reduction of Perceived Stress sources as an intervention goal.

### 4.3 The mediating role of psychological distress between perceived stress and mental health

The study’s findings reveal that psychological distress plays a significant mediating role in the link between perceived stress and psychological symptom severity. This confirmed our research hypothesis H2. This finding is in line with other studies that observed the psychological mechanisms during the pandemic. For example, Zhang et al. found that psychological distress is an important intermediate variable in the relationship between stress and depression (51);At the same time, the research by Lara-Cabrera and others revealed a strong link between perceived stress and mental health problems such as anxiety and depression (52), which indirectly supports the idea that stress needs to be addressed by affecting internal psychological reactions to have a positive effect on overall mental health;Sudhakar et al. (53) also found that stress-related sexual events can exacerbate an individual’s anxiety and depression, thereby leading to a decline in overall psychological well-being. This finding can be interpreted through the lens of Response Style Theory, which suggests that when perimenopausal women are faced with intense stress, they are more likely to fall into a maladaptive cognitive pattern known as rumination, whereby they passively and repeatedly dwell on their own negative emotions and stressors (22). This repetitive negative thinking directly maintains a state of Psychological distress within, which is itself a core manifestation of impaired mental health, thus constituting a mediating pathway between stress and health outcomes. The validity of this mediating mechanism is supported by intervention studies. For example, a randomized controlled trial involving perimenopausal women found that an 8-week mindfulness-based stress reduction program significantly reduced participants’ depressive symptoms, perceived stress, and anxiety levels (54). The study further indicated that one mechanism of action for such mindfulness interventions involves reducing maladaptive cognitive responses, such as rumination and worry. This experimental evidence strengthens the causal inference that mindfulness training can effectively mitigate the negative impact of stress on psychological symptom severity by blocking maladaptive cognitive responses. Based on these findings, it is recommended that when implementing interventions for perimenopausal women’s mental health, the reduction of psychological distress should be the primary intervention goal. Cognitive-behavioral therapy (55, 56), for example, can be used to identify and change ruminative thinking and develop positive coping strategies.

### 4.4 The mediating role of perceived social support between perceived stress and mental health

The study’s findings suggest that perceived social support acts as a mediator between perceived stress and psychological symptom severity. This finding validates hypothesis H3, demonstrating that positive social support mitigates the impact of stress on health outcomes. This discovery aligns with results from other studies (5, 57, 58). The buffering model explains this mediating effect by highlighting the significant role of social support in helping individuals cope with stressful events (30). High levels of perceived Stress diminish an individual’s appraisal of the availability of his or her social support network, and when an individual feels that he or she lacks sufficient understanding, care, and practical help, he or she has fewer psychological resources to cope with the stress, and is more likely to experience a sense of hopelessness, which can exacerbate deterioration of mental health (58). The study indicates that for perimenopausal women, stress predominantly affects mental health by directly influencing internal emotional states, with social support serving as an additional protective factor due to the pathway’s relatively small effect size. This discovery suggests that mental health strategies targeting this population should focus not only on addressing stressors themselves but also on strengthening and maintaining their social support systems. Interventions designed to enhance social support have been shown to be effective in buffering stress. Support for this comes from an intervention study involving menopausal women. This study implemented a six-month composite intervention incorporating key social support components, such as forming peer support groups, encouraging experience sharing, and providing ongoing informational support (35). Compared to the control group, women receiving the intervention showed significant improvements in stress-related psychological symptoms like depression and anxiety. Although the study did not directly measure changes in perceived social support, its intervention design embedded theoretical and practical elements of social support. The observed improvements align with the theoretical pathway of buffering stress through enhanced support networks. This provides indirect yet reasonable empirical support for establishing or strengthening social support networks as an intervention target for alleviating stress-related emotional issues in middle-aged women. When developing interventions, the specific cultural context of China must be fully considered. Confucian culture emphasizes family harmony, filial piety, and collective values. While these principles constitute a potentially significant source of social support, they may also attach expectations of reciprocal obligations to such support. Furthermore, women may hesitate to actively seek support due to concerns about “saving face.” Therefore, for generic supportive activities (such as family relationship workshops or peer support groups) to achieve maximum effectiveness, they must incorporate culturally adapted psychological education. This education should guide family members to understand “providing emotional support” as a shared responsibility for maintaining the overall health of the family unit, rather than merely a burden for addressing individual emotional issues. Simultaneously, skill training should help women seek and utilize social support more effectively in stressful situations. Only through such comprehensive interventions can we fundamentally mitigate stress’s erosion of perceived social support, thereby more effectively blocking the pathway through which stress impacts psychological symptom severity.

### 4.5 The mediating role of psychological distress and perceived social support in the chain of effects between perceived stress and mental health

The study confirmed that psychological distress and perceived social support significantly mediate the relationship between perceived stress and psychological symptom severity in a sequential manner. This result confirms Hypothesis H4 and is consistent with Pearlin’s stress process model (36). The plausibility of this chained mechanism is supported by evidence from its constituent pathways. First, the association between perceived stress and increased psychological distress has been widely established, and our data further corroborate this finding (24, 25). Second, research indicates that psychological distress subsequently weakens perceived social support, as negative emotional states may lead to social withdrawal and reduced help-seeking behaviors (38). This study integrates these two established pathways, testing their sequential operation as a coherent chai within the SPM framework for this specific population. Our analysis provides a crucial quantitative evaluation of this chain. Although the intermediate pathway was statistically significant, it only accounted for 3.62% of the total effect. This suggests that, although the sequential process was a novel theoretical discovery in this study population, it had a limited actual contribution to the overall variation in the severity of psychological symptoms compared to the direct effect of stress and other intermediate pathways. This finding refines the application of the SPM model, indicating that while the chain from distress to support erosion is effective, its quantitative contribution is relatively minor. Consequently, the primary contribution shifts from merely confirming the chain’s existence to understanding its relative magnitude within broader stress processes.This refined interpretation guides intervention practices. For perimenopausal women, interventions should prioritize alleviating direct stressors and reducing psychological distress. Simultaneously, for individuals experiencing significant distress, strategies should proactively support their social networks (e.g., through social skills training or family psychoeducation) to prevent secondary erosion of potential support resources, thereby forming more comprehensive intervention plans.

## 5 Limitations and shortcomings

This study initially uncovered how perceived stress affects psychological symptom severity of perimenopausal women via psychological distress and perceived social support. It still has the following limitations: (1) This study used a cross-sectional design, collecting all data at a single time point. This design cannot provide strong evidence for causal relationships between variables. Future studies should use longitudinal designs to track the dynamic evolution of these social psychological factors during the menopausal transition. Key time points to track include the initial appearance of menstrual cycle irregularities (early menopause), last menstrual period, and the first and second years after menopause. Measuring perceived stress, psychological distress, social support, and mental health at these critical transition points will greatly improve our understanding of causal pathways and critical intervention windows; (2) All core variables were measured using self-report questionnaires. Although these scales exhibit strong reliability and validity, the method may be influenced by social desirability effects and recall bias, possibly overstating the associations between variables. Future studies could incorporate objective physiological indicators to enhance the objectivity of findings; (3) This study employed convenience sampling, with participants primarily drawn from a specific community and consisting solely of Chinese perimenopausal women. This limits the generalizability of findings. The sample may lack sufficient representativeness, failing to adequately encompass women from diverse geographic regions and socioeconomic backgrounds. Future research should focus on validating findings through probability sampling methods that enhance national representativeness.

## 6 Conclusion

This study explores how psychological distress and perceived social support mediate the link between perceived stress and psychological symptom severity in perimenopausal women. The study reveals that psychological distress and perceived social support independently mediate the link between perceived stress and psychological symptom severity, while also creating a sequential mediation pathway from stress perception to symptom severity outcomes. Based on these findings, to reduce psychological symptom severity and thereby improve overall psychological symptom severity and thereby improve mental health interventions for perimenopausal women can be approached in two ways: directly addressing stressors to help them develop effective stress management strategies that reduce perceived stress; and alleviating psychological distress through timely psychological counseling while enhancing the accessibility of coping resources by strengthening social support networks. Simultaneously, preventing and mitigating excessive stress perception at its source will also indirectly reduce symptom burden.

## Data Availability

The original contributions presented in the study are included in the article/**Supplementary Material**. Further inquiries can be directed to the corresponding author.
